# Histological criteria for atypical pituitary adenomas – data from the German pituitary adenoma registry suggests modifications

**DOI:** 10.1186/s40478-015-0229-8

**Published:** 2015-08-19

**Authors:** Christian P. Miermeister, Stephan Petersenn, Michael Buchfelder, Rudolf Fahlbusch, Dieter K. Lüdecke, Annett Hölsken, Markus Bergmann, Hans Ulrich Knappe, Volkmar H. Hans, Jörg Flitsch, Wolfgang Saeger, Rolf Buslei

**Affiliations:** Departments of Neuropathology, Friedrich-Alexander University Erlangen-Nürnberg (FAU), Schwabachanlage 6, 91054 Erlangen, Germany; ENDOC Center for Endocrine Tumors, Hamburg & University of Duisburg-Essen, Essen, Germany; Departments of Neurosurgery, Friedrich-Alexander University Erlangen-Nürnberg (FAU), Erlangen, Germany; Department of Neurosurgery, International Neuroscience Institute, Hannover, Germany; Departments of Neurosurgery, University Clinic Hamburg-Eppendorf, Hamburg, Germany; Department of Neuropathology, Klinikum Bremen Mitte, Bremen, Germany; Department of Neurosurgery, Johannes Wesling Hospital Minden, Minden, Germany; Department of Pathology, Ruhr University Bochum, Bochum, Germany; Departments of Neuropathology, University Clinic Hamburg-Eppendorf, Hamburg, Germany

## Abstract

**Introduction:**

The term *atypical pituitary adenoma* (APA) was revised in the 2004 World Health Organization (WHO) classification of pituitary tumors. However, two of the four parameters required for the diagnosis of APAs were formulated rather vaguely (i.e., *“extensive”* nuclear staining for p53; *“elevated”* mitotic index). Based on a case-control study using a representative cohort of typical pituitary adenomas and APAs selected from the German Pituitary Tumor Registry, we aimed to obtain reliable cut-off values for both p53 and the mitotic index. In addition, we analyzed the impact of all four individual parameters (invasiveness, Ki67-index, p53, mitotic index) on the selectivity for differentiating both adenoma subtypes.

**Methods:**

Of the 308 patients included in the study, 98 were diagnosed as APAs (incidence 2.9 %) and 10 patients suffered from a pituitary carcinoma (incidence 0.2 %). As a control group, we selected 200 group matched patients with typical pituitary adenomas (TPAs). Cut-off values were attained using ROC analysis.

**Results:**

We determined significant threshold values for p53 (≥2 %; AUC: 0.94) and the mitotic index (≥2 mitosis within 10 high power fields; AUC: 0.89). The most reliable individual marker for differentiating TPAs and APAs was a Ki-67-labeling index ≥ 4 % (AUC: 0.98). Using logistic regression analysis (LRA) we were able to show that all four criteria (Ki-67 (*p* < 0.001); OR 5.2// p53 (*p* < 0.001); OR 3.1// mitotic index (*p* < 0.001); OR 2.1// invasiveness (*p* < 0.001); OR 8.2)) were significant for the group of APAs. Furthermore, we describe the presence of nucleoli as a new favorable parameter for TPAs (*p* = 0.008; OR: 0.4; CI95 %: 0.18; 0.77).

**Conclusions:**

Here we present a proposed rectification of the current WHO classification of pituitary tumors describing an additional marker for TPA and specific threshold values for p53 and the mitotic index. This will greatly help in the reliable diagnosis of APAs and facilitate further studies to ascertain the prognostic relevance of this categorization.

**Electronic supplementary material:**

The online version of this article (doi:10.1186/s40478-015-0229-8) contains supplementary material, which is available to authorized users.

## Introduction

**Fig. 1:**
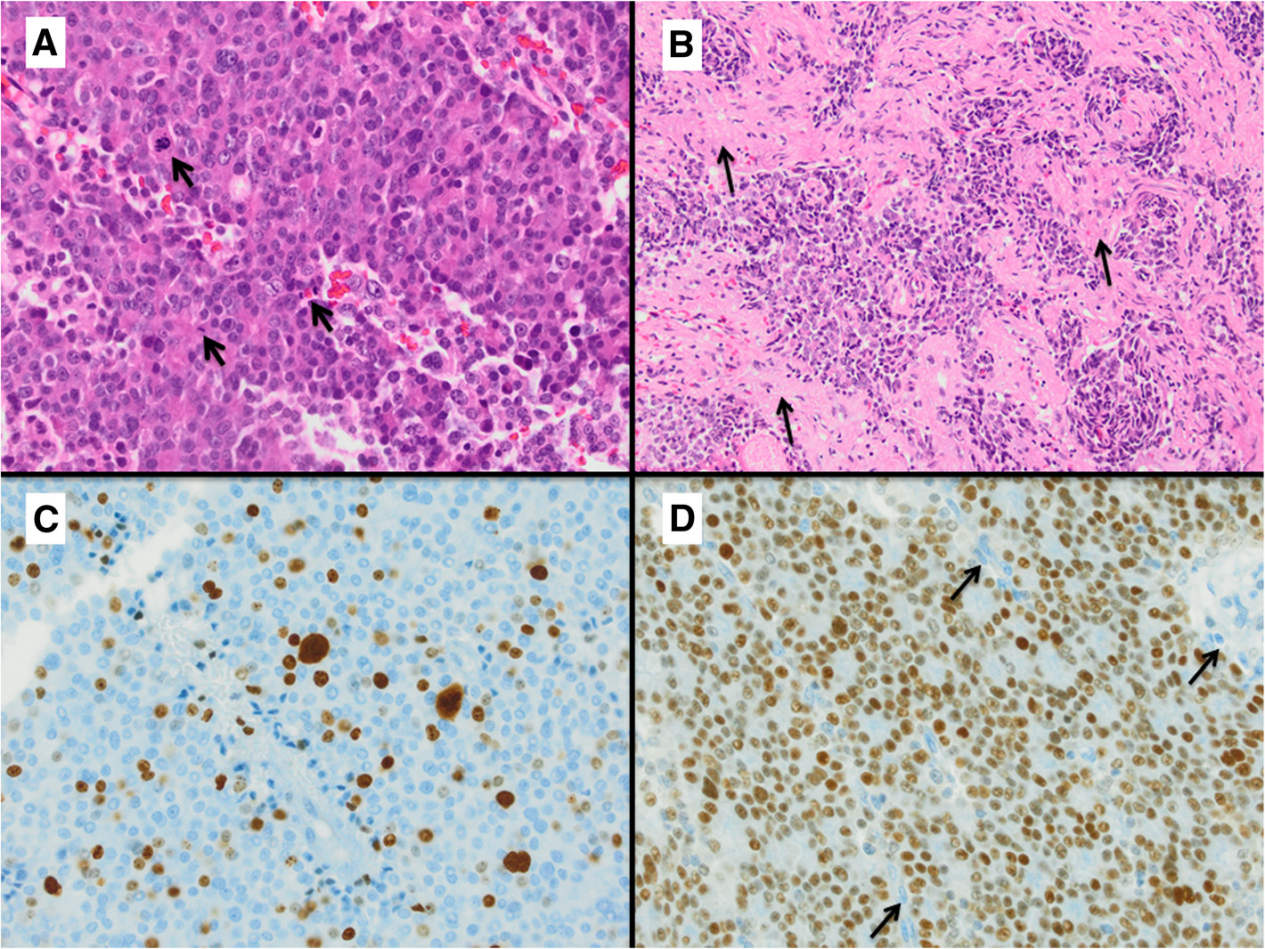
Diagnostic criteria for atypical pituitary adenomas. An example of an atypical pituitary adenoma (ACTH-cell adenoma) with several mitotic figures (Arrows in **a**) in one HPF (HE staining, magnification 400x), infiltration of surrounding meninges (Arrows in **b**, HE staining, magnification 100x), Ki67 index >4 % (**c**, magnification 400x) and strong nuclear p53 expression in >3 % of cells (**d**, magnification 400x; Arrows: negative endothelial cells indicating antibody specificity)

Pituitary adenomas (PAs) are the most common benign neoplasms in the sellar region, occurring in 10 % [1] to 20 % [2, 3] of the general population. In most cases, they represent slowly growing, clinically nonfunctioning tumors developing from adenohypophysial cells [4]. Earlier classification systems were based on tumor size (microadenomas <10 mm vs. macroadenomas >10 mm) and basic staining characteristics (acidophilic, basophilic, chromophobic). Today, histopathological analysis of the hormone expression profile using immunohistochemistry allows for the differentiation of several subtypes and variants (e.g., GH, PRL, ACTH, TSH, FSH, LH, plurihormonal, null cell adenomas, densely and sparsely granulated tumors) [5, 6]. For prognostic purpose, the current 2004 WHO classification of tumors of endocrine organs revised diagnostic criteria for the group of atypical pituitary adenomas (APAs). The aim was to identify tumors with histomorphological signs of intermediate malignancy, most likely indicating uncertain clinical and biological behavior. Furthermore, APAs were thought to be the precursor lesion of the very rare group of pituitary carcinomas (PCA), representing the only malignant primary sellar tumor entity (0.2 %) which per definition featured systemic and/or cerebrospinal metastases. Histological and immunohistochemical criteria were defined for the diagnosis of APA: 1.) Invasive tumor growth; 2.) Ki-67 labeling index (LI) greater than 3 %; 3.) Elevated mitotic activity; 4.) Extensive nuclear staining for p53 [7, 5] (Fig. 1). In comparison to existing diagnostic criteria for other primary brain tumors with intermediate malignancy such as atypical meningiomas, some of the criteria (especially p53 and the mitotic index), were formulated rather vaguely. This may be one explanation for the different frequencies of APAs published in several larger series since 2004 [8, 6, 9]. To address this important issue and to increase diagnostic clarity and reproducibility in routine diagnostic work, we initiated a case-control study using a large cohort of 308 patients selected from the German Pituitary Tumor Registry. Specific to this registry, all samples were analyzed by only two different pathologists with a long-standing expertise in the investigation of pituitary tumors (WS and RB). Cases sent to the register were documented and the initial diagnoses were reviewed using new slides and stainings. Two great benefits of this approach are the avoidance of inter-observer heterogeneity and the standardization of both technical and analytical processes. A reliable and reproducible diagnosis is the basis for initiating further studies to clarify the justification of the diagnosis APA as an own subgroup of pituitary tumors.

## Materials and methods

### Patient collective

Patients were identified from the German Pituitary Tumor Registry in cooperation with members of the German pituitary working group (see Acknowledgements). Pathological reports from a total of *N* = 4232 patients documented between 2005 and 2012 were analyzed. Therefore, 4101 tumors were diagnosed as typical pituitary adenomas (TPAs; 96.9 %) and 121 as atypical pituitary adenomas (APAs; 2.9 %). A group of ten patients with a pituitary carcinoma (PCA; 0.2 %) diagnosed between 1995 and 2011 was also included. The inclusion criteria for statistical analysis was the presence of a minimum of three of the parameters suggested by the WHO for the diagnosis of APAs: *p53 immunoreactivity, MIB-1 (Ki-67) index, mitotic activity and invasiveness*. Each marker was analyzed for each group separately. Twenty three APAs had to be excluded due to incomplete clinical and/or histopathological data or resulting from the absence of two or more of the aforementioned criteria. However, a total of 98 cases did meet the requirements and were finally selected for the study. Moreover, 200 group matched patients (in terms of age, sex and adenoma subtype) with TPAs served as a control group [10]. Overall we analyzed a cohort of 98 APAs, 10 PCAs and 200 group matched TPAs (Table 1).

**Table 1:** Histological tumor classification

Tumour subtypes	Study groups							
	TPAs		APAs		PCAs		Total	
	Number	%	Number	%	Number	%	Number	%
Densely granulated GH-cell adenomas	5	2.5 %	3	3.1 %	0	0.0 %	8	2.6 %
Sparsely granulated GH-cell adenomas	16	8.0 %	11	11.2 %	1	10.0 %	28	9.1 %
Mixed GH/PRL-cell adenomas	9	4.5 %	6	6.1 %	0	0.0 %	15	4.9 %
PRL-cell adenomas	64	32.0 %	32	32.7 %	2	20.0 %	98	31.8 %
Densely granulated ACTH-cell adenomas	19	9.5 %	8	8.2 %	0	0.0 %	27	8.8 %
Sparsely granulated ACTH-cell adenomas	49	24.5 %	20	20.4 %	0	0.0 %	69	22.4 %
ACTH-cell adenomas, NOS	0	0.0 %	0	0.0 %	7	70.0 %	7	2.3 %
TSH-cell adenomas	3	1.5 %	2	2.0 %	0	0.0 %	5	1.6 %
FSH/LH-cell adenomas	10	5.0 %	5	5.1 %	0	0.0 %	15	4.9 %
Null cell adenomas	25	12.5 %	11	11.2 %	0	0.0 %	36	11.7 %
Total	200	100 %	98	100 %	10	100 %	308	100 %

The histological classification of each tumor analyzed in the groups of typical pituitary adenomas (TPA), atypical pituitary adenomas (APA) and pituitary carcinomas (PCA) is shown in detail. *NOS* not otherwise specified

*GH* growth hormone, *PRL* prolactin, *ACTH* adrenocorticotrophic hormone, *TSH* thyroid-stimulating hormone, *FSH* follicle-stimulating hormone, *LH* luteinizing hormone

Patient age at surgery, gender, as well as histopathological tumor parameters such as immunohistochemical hormone expression (GH, PRL, ACTH, TSH, FSH, LH, α-subunit), protein S100 expression, presence of nucleoli and invasiveness, mitotic activity and expression of the cell cycle markers p53 and Ki-67 were recorded (Table 1 and Table 3). All of the cases were stained twice, once by the initial pathologist and once by the registry lab and the number of positive cells was determined in 10 representative high power fields during the initial diagnosis and the re-evaluation process. Only nuclei with a distinct nuclear expression were taken into account (see Fig. 1). In cases of inter-observer heterogeneity, a second and third evaluation were conducted. Verification of tumor invasion in surrounding anatomical structures (e.g., meninges, bone, brain tissue, sphenoidal sinus) was evaluated using either surgical reports, the preoperative MRI samples or confirmed by clear histology.

### Tumor specimens and staining results

All specimens were routinely formalin fixed, embedded in paraffin and stained with hematoxylin-eosin and PAS-reaction. The following immunostainings were performed for each sample: anti-GH (monoclonal), Novocastra, Newcastle upon Tyne, UK, 1:1000; anti-Prolactin (monoclonal), Zytomed, Berlin, Germany, 1:12000, anti-ACTH (polyclonal), Zytomed, Berlin, Germany, 1:30; anti-TSH (monoclonal), Immunotech, Marseille, France, 1:5000, anti-FSH (monoclonal), Immunotech, Marseille, France, 1:20000, anti-LH (monoclonal) Immunotech, Marseille, France, 1:2500, anti-alpha-subunit (monoclonal), Immunotech, Marseille, France, 1:1500, anti-Ki67/MiB-1 (monoclonal), Zytomed, Berlin, Germany, 1:750, anti-p53 (monoclonal) Novocastra, Newcastle upon Tyne, UK, 1:150, anti-S100-protein (polyclonal) Dako, 1:1250, and for differentiation of sparsely and densely granulated GH cell adenomas anti-Keratin/KL1 (monoclonal), Immunotech, Marseille, France, undiluted.

Both Ki-67 labeling index (LI) and the number of p53 immunopositive nuclei were independently, semi-quantitatively assessed by two experienced pathologists (WS and RB) within hot spot areas of the tumor samples. Mitotic figures were retrospectively quantified within ten representative high power fields (HPF of 0.30 mm^2^, 400 × magnification) using hematoxylin and eosin stained (H&E) sections by two investigators (CM and RB), in all samples evaluable (APAs *n* = 78, TPAs *n* = 151 and PCAs *n* = 4). The existence of nucleoli was evaluated in the same way in a cohort of 77 APAs, 148 controls and 7 PCAs.

### Study design and statistical analysis

In order to show strength of influence, we applied logistic regression analysis (LRA) to the combination of the currently proposed markers for atypia independently (Ki-67 LI, mitotic rate, p53 expression, invasiveness; according to the WHO classification system of endocrine tumors) in a large cohort of TPAs (“controls”), APAs (“cases”) and PCAs (“cases”) [11]. Receiver operator curve (ROC) analyses were performed for each parameter to find reliable cut-off values, and the Youden index, as well as area under curve (AUC) served as quality control [12, 13]. The AUC values were interpreted as follows: 0.5–0.7 = minimal; 0.7–0.9 = moderate; >0.9 = high discriminatory power [14, 13]. We compared the groups of APAs and PCAs versus Controls as well as PCAs and APAs respectively to evaluate the individual impact and significance of each marker. Three additional markers (α-subunit, protein S100, nucleoli) were studied in subgroups (APA/TPA) and their properties were analyzed using LRA to investigate their relevance as diagnostic factors on their own. Furthermore, additional odds ratios (OR) were calculated for each parameter. “An odds ratio (OR) is a measure of association between an exposure and an outcome. The OR represents the probability that an outcome will occur given a particular exposure, compared to the odds of the outcome occurring in the absence of that exposure. Odds ratios are most commonly used in case-control studies (…) [15].” The pseudo-coefficient of determination (Nagelkerkes R^2^) was used to measure the predictive power of the model [16, 17].

The correlations between individual metric parameters (Ki-67, p53, mitosis) were analyzed by Spearman’s rank correlation. In case of the dichotomous parameter invasiveness, the point-biserial correlation coefficient was assessed [18]. Additionally, the correlation between the subgroups and metric parameters were also calculated using the point-biserial method. The phi coefficients were used for dichotomous (invasiveness, nucleoli) and ordinal coded data (α-subunit, protein S100) related to the subgroups. With respect to ordinal coded data, the results were verified using Cramers V [18]. The accuracy was calculated in common way $\left( accuracy=\frac{number\  of\  true\  positives+ number\  of\  true\  negatives}{number\  of\  true\  positives+ false\  positives+ false\  negatives+ true\  negatives}\right)$ [19]. *P*-values less than 5 % were viewed as being statistically significant and for all statistical analyses IBM SPSS Statistics 21 software was used.

## Results

### Clinical characteristics

Out of the 308 patients included in the study, 98 were diagnosed as atypical pituitary adenomas (APAs). A typical example is presented in Fig. 1, showing mitotic figure, high levels of Mib-1 and p53, as well as invasion of surrounding structures. This group was composed of 51 men (52 %, mean age at surgery = 42 years, range 16–78 years) and 46 women (47 %, mean age at surgery = 46 years, range 13–83 years). In one case, the gender was not documented. The tumors correspond to thirteen different adenoma subtypes (including sparsely and densely granulated variants; Table 1) of which the largest group were diagnosed as prolactin cell adenomas (*n* = 32; 32.7 %) followed by: ACTH-cell adenomas (*n* = 28; 28.6 %), GH-cell adenomas (*n* = 14; 14.3 %), null-cell adenomas (*n* = 11; 11.2 %), mixed GH/Prolactin cell adenomas (*n* = 6; 6.1 %), FSH/LH-cell adenomas (*n* = 5; 5.1 %) and TSH-cell adenomas (*n* = 2; 2 %) 12 of these cases (12.1 %) were relapses.

The case study group and the control group (*n* = 200 patients) had equivocal comparative values with regards to age and gender. One particular patient is listed in both, the case study group (surgery in 2009) and also in the PCA group (relapse 2010), but this case was not applied for statistical analysis.

The group of patients with pituitary carcinomas (PCAs) consists of six men (60 %; mean age 40; range 24–53 years) and four women (40 %; mean age 61; range 53–77years). Seven patients included in the study suffered from an ACTH-cell carcinoma (70 %), two patients had a PRL-cell carcinoma (20 %) and one patient a sparsely granulated GH-cell carcinoma (10 %). Detailed clinical data is summarized in Table 1.

### ROC analysis

**Fig. 2:**
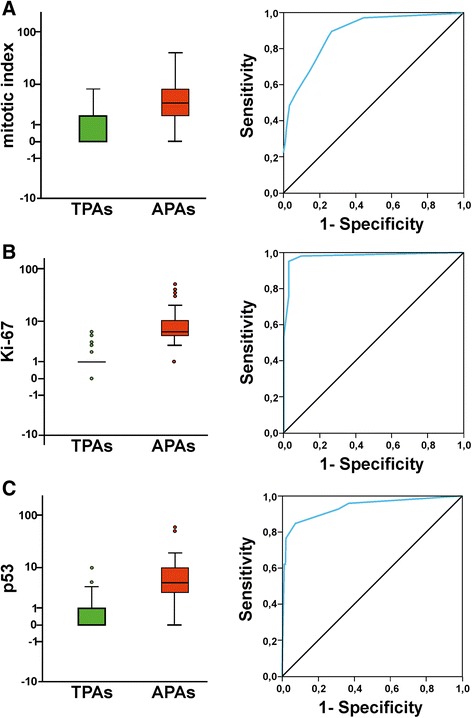
Statistical analysis of the mitotic index, Ki-67 and p53 in the groups of TPA and APA. The Boxplot and Receiver operator curve (ROC) analysis based on the mitotic index (=number of mitosis counted in 10 high power fields (HPF); 400x magnification; **a**) and the values of tumor cell nuclei showing a distinct Ki-67 (**b**) and p53 (**c**) expression (%) in hot spot areas of the single tumor samples. The Y-axis is log scaled to figure out a clearer illustration of the lower values. Single outliers are presented as circles. The range and distribution of the analyzed parameters Ki67, p53 and number of mitotic figures in both subgroups (TPAs/APAs) is visualized using box plots. The boxes represent the 25th and the 75th percentiles, including the median value as a dark horizontal bar

ROC analysis determined a cut-off ≥ 2 Mitoses in 10 HPF for the number of mitoses. There was a range of 0–8 mitoses among the group of TPAs and a range of 0–41 in the group of APAs respectively. Sensitivity was 90 % and specificity 74 %. The quality of the diagnostic tests was determined with a Youden index rating of 0.64 and an AUC of 0.89 (Fig. 2a). Therefore, accuracy is up to 79 %. The identified threshold value for the MIB-1 proliferation index of ≥4 % was slightly higher than the current cut-off value suggested by the WHO (>3 %). The spectrum of Ki-67 LI for TPAs ranged from 0 to 6 % and for the group of APAs between 1 and 50 %. Sensitivity was 95 % and specificity 97 %. The good quality of these diagnostic tests was confirmed by the Youden index value of 0.92 and the AUC of 0.98 (Fig. 2b). Accuracy was scored at 96 %. A distinct nuclear staining in ≥ 2 % of cells was found to be the best cut-off value for p53. The span of the controls ranged from 0 to 10 % and from 0 to 60 % in the group of APAs. Sensitivity and specificity were found at 85 % and 93 % respectively (Youden index: 0.78). With an AUC of 0.94, a high discriminatory power was evident (Fig. 2c). The accuracy in this case was 90 %. The entire data is presented in Table 2 and furthermore an overview of the average values from mitosis, Ki-67 and p53 is shown in Table 3.

**Table 2:** Statistical values of the main histomorphological parameters

Diagnostic Parameters for APAs/TPAs	Cut-off	Sensitivity	Specitifity	Youden-Index	Accuracy in %	AUC	95 % CI of AUC	OR	95 % CI of OR	*P*-value
Ki-67 pos. nuclei in %	≥4	0.95	0.97	0.92	96	0.98	[0.96; 1.0]	5.2	[3.43; 7.83]	<0.001
P53 pos. nuclei in %	≥2	0.85	0.93	0.78	90	0.94	[0.90; 0.97]	3.1	[2.31; 4.04]	<0.001
Mitotic Index in 10 HPF	≥2	0.90	0.74	0.64	79	0.89	[0.84; 0.93]	2.1	[1.70; 2.57]	<0.001
Invasiveness	Yes	0.88	0.53	0.41	64	-	-	8.2	[3.66; 18.42]	<0.001

The proposed threshold values for Ki-67, p53, number of mitotic figures in 10 HPF (high power fields) and the status of invasive tumor growth, to distinguish APA and TPA are shown with their respective statistical values

*OR* odds ratio, *AUC* area under curve, *CI* confidence interval

**Table 3:** Frequency of WHO criteria in the different tumor subtypes

		Subgroups						All Patients	
		TPAs		APAs		PCAs		Total	
		N=	Mean	N=	Mean	N=	Mean	N=	Mean
Mitotic index (in 10 HPF)		200	1	98	7	10	3	308	3
p53 pos. Nuclei (%)		200	1	98	8	10	8	308	3
Ki-67 pos. Nuclei (%)		200	1	98	9	10	7	308	4
Growth pattern	Non invasive	77	-	8	-	0	-	85	-
	Invasive	68	-	58	-	10	-	136	-

Overview of metric and dichotomous parameters analyzed in the subgroups of pituitary tumors analyzed

*N* number, *HPF* High power field, *TPA* typical pituitary adenomas, *APA* atypical pituitary adenomas, *PCA* pituitary carcinomas

### Reliability of markers

Using a binary logistic regression analysis (LRA), it was verified that all four predictors (invasiveness, mitotic rate, p53, Ki-67) significantly contributed to the definition of the dependent variables (typical/atypical adenoma). An error reduction (“pseudo-coefficients of determination”) according to Nagelkerke’s R^2^ of 0.86 for Ki67, 0.69 for p53, 0.53 for the number of mitosis and 0.22 for invasiveness was calculated, acknowledging the associated predictive power. Therefore, it can be said that the existence of an APA increases by a factor of 8.2 (*p* < 0.001) when an invasive growth pattern is present (Sensitivity 88 %, Specificity 53 %, Youden Index 0.41, Accuracy 64 %), by a factor of 5.2 (*p* < 0.001) per percentage point of Ki67 positive tumor cell nuclei, by a factor of 3.1 (*p* < 0.001) with each percentage point of p53 immunopositive nuclei and with a factor of 2.1 (*p* < 0.001) per every single mitosis in 10 HPF (Table 2; Fig. 2a, b, c).

Taking only into account the newly suggested Ki67 (≥4 %) and p53 (≥2 %) cut off values, it was possible to correctly categorize 286/298 tumors (7 false pos. & 5 false neg.) and 268/298 tumors (15 false pos. & 15 false neg.) respectively. A combination of both parameters enabled for the right diagnosis of 277/298 samples (21 false pos. & 0 false neg.). The correlation analysis showed that all four parameters discussed were significant amongst themselves as well as in relation to the subgroups (typical/atypical) on the level 0.001. Only invasiveness and p53 were significant on level 0.05 in relation to each other. A positive correlation coefficient was detected in both models. Statistical data is summarized in Additional file 1: Table S1 and Additional file 2: Table S2.

Neither the correlation analysis nor the LRA showed significant differences between the *case study and the control group* with respect to protein S100 (*p* = 0.151, r_phi_ = 0.134; LRA: *p* = 0.269, R^2^ = 0.006) and the α-subunit (*p* = 0.138, r_phi_ = 0.137; LRA: *p* = 0.955, R^2^ = <0.001). However, both models showed significant differences between the two subgroups with regard to existence of nucleoli (*p* = 0.006, r_phi_ = −0.181; LRA: *p* = 0.008, R^2^ = 0.04). A low negative correlation coefficient was measured. It must be noted that the presence of nucleoli reduced the risk of an APA by a factor of only 0.4 (*p* = 0.008; CI95 %: 0.18; 0.77, R^2^ = 0.04).

### Pituitary carcinomas

Ten PCAs were compared to 40 TPAs of the control group. The latter were selected on the basis of age, sex and adenoma subtype. The cut-off values, which were determined in the group of APA cases, were applied to the parameters Ki-67, p53 and the mitotic index, without prior ROC analysis due to the small number of samples available. The overall incidence of PCAs in our series of sellar tumors was 0.2 %. Point-biserial analysis showed that Ki-67 (*p* < 0.001; r_pb_ = 0.556), p53 (*p* < 0.001; r_pb_ = 0.483) and the phi coefficient of invasiveness (*p* = 0.004; r_phi_ = 0.439) were significant at the 0.01 level in correlation with both subgroups (PCA/TPA). A positive correlation coefficient was detected in these cases. The mitotic index reached no significant level (*p* = 0.097; r_pb_ = 0.266), but yet attained a sensitivity of 100 %. Only four of ten PCAs could be reevaluated with respect to the number of mitoses. Statistical data is summarized in Additional file 1: Table S1.

The LRA showed that only the parameters Ki-67 (OR: 1.8) (*p* = 0.012; R^2^ = 0.41) and p53 (OR: 1.8) (*p* = 0.01; R^2^ = 0.44) were significant regressors, whereas number of mitosis (*p* = 0.124; R^2^ = 0.11) and invasiveness (*p* = 0.998; R^2^ = 0.36) failed this assumption. A sensitivity of 60 % and a specificity of 93 % as well as an accuracy of 86 % were obtained for p53 and Ki67. Due to matching 12 TPAs were not taken into account with regard to mitosis (*n* = 4) and invasiveness (*n* = 8).

In contrast, another model showed neither significant differences between PCAs (*n* = 10) and APAs (*n* = 20) with respect to the markers Ki-67 (*p* = 0.223), p53 (*p* = 0.585), the mitotic index (*p* = 0.274) and invasiveness (*p* > 0.999) featured in the LRA, nor in the point-biserial correlation analysis with values of Ki-67 (*p* = 0.204), p53 (*p* = 0.594), mitotic index (*p* = 0.189) and the phi coefficient of invasiveness (*p* = 0.245). Statistical data is summarized in Additional file 1: Table S1. Due to matching, six APAs were not taken into account with regard to mitosis (*n* = 2) and invasiveness (*n* = 4).

## Discussion

The goal of this study was to further specify the vaguely described histomorphological and immunohistochemical parameters for the diagnosis of an atypical adenoma (APA). Even though this diagnosis was introduced more than ten years ago by the World Health Organization (WHO), specific cut-off values for the criteria “elevated mitotic index” and “extensive nuclear staining for p53 immunoreractivity” are still missing. Furthermore, we tested the consistency of the four suggested criteria for atypical tumor growth (i.e., Ki-67, invasiveness, number of mitosis and p53 levels) [7, 20–24, 6, 25], comparing a large cohort of typical pituitary adenomas (TPAs), APAs and pituitary carcinomas (PCAs). All the tumor cases were selected from the German Pituitary Tumor Registry in Hamburg. During the time period examined (2005–2012), APA reached an overall frequency of 2.9 % (121/4231), a value which increased only slightly compared to the frequency of 2.7 % (12/451) described in 2007 [6]. In line with previously published data from 2007, more than 84 % (*n* = 83/98) of APAs can be classified as either sparsely granulated prolactinomas, ACTH secreting adenomas, growth hormone producing adenomas or null cell adenomas [6]. A study of Zada et al. published in 2011 [8] showed a similar subtype distribution within the group of APAs, but a clearly higher occurrence of 14.9 % (*n* = 18/121). This was confirmed by another group describing an incidence value of 8.9 % (*n* = 13/146), respectively [9]. These varying frequencies may reflect the problems in using the existing diagnostic criteria for APAs, irrespective of the experience of the pathologist. Thus the first important aspect of the study presented here was to suggest reliable, reproducible and easy to predict cut-off values for the mitotic rate and the p53 expression level.

Using ROC curve analysis, we defined a valid cut-off value for the number of mitotic figures as ≥ 2 mitoses per 10 HPF in APA cases. The quality of the chosen threshold value is documented by a Youden index rating of 0.64 and an AUC of 0.89, as well as a sensitivity of 90 % and a specificity of 74 %. Overall, this single parameter allows a correct graduation in up to 79 % of cases which indicates that it should not be used alone when making a reliable diagnosis. Despite the difficulties attempting to choose a correct cut-off value, the risk of an APA increases by a factor of 2.1 (*p* < 0.001) per each mitosis within 10 HPF (Table 3). In general, the number of mitosis are easy to achieve although tissue shrinkage, delayed fixation time and bleeding may cause problems and should be taken into account [22].

Nuclear accumulation of p53 as a prognostic marker for pituitary tumors is discussed controversially throughout the literature. There are several studies featuring different results with regard to its importance in the growth behavior (aggressive/invasive) of adenomas [26–32, 21, 33–36]. Using our large cohort and the analytical, statistical and technical methods as described, we propose using a threshold value for p53 of ≥2 % of clearly immunoreactive nuclei for the diagnosis of an APA. More than 93 % of the control cases showed a lower expression (<2 %) (Table 3). The very high specificity value (93 %) proves that p53 protein expression ≥2 % (Youden index 0.78) is an extremely helpful and significant parameter. Nevertheless, even an absolute negative staining result does not eliminate the possibility of aggressive/invasive tumor growth, indicated by a relatively low sensitivity (85 %). The good quality of the p53 value, independent from the cut-off, is further supported by the AUC (0.94). It must be taken into account that the immunohistochemical detection of p53 is dependent upon on the antibody and the method used for investigation [37].

In addition to describing detailed cut-off values for p53 immunoreactivity and the number of mitotic figures, we further analyzed the discriminatory power of the existing Ki67 labeling index and an invasive tumor growth pattern. The latter was propagated as a helpful marker to distinguish TPAs from APAs and was already mentioned in the previous WHO classification system [5]. The importance of measuring the Ki-67 LI with respect to invasiveness, progression and clinical characteristics is highly controversial [38–46, 36, 47]. The varying counting methods and antibodies used to determine the K67 LI may be, in part, an explanation for the contradictory results already published [42, 47]. Although both levels mathematically represent the same group of samples (>3 % = ≥4 %), we would prefer using a cut-off value for Ki-67 ≥ 4 % for our cohort as this has the better discriminatory power and is more precisely defined (Table 3). A very high specificity (97 %) and sensitivity (95 %) indicates indeed that the proliferation index is a very good and reliable diagnostic tool (Youden index 0.92) that provides important results for the diagnosis of an APA. In comparison to p53 immunoreactivity (0.94) and the mitotic index (0.89), the Ki-67 LI had the highest AUC (0.98), suggesting it to be the best single parameter for the diagnosis of APAs, which is in line with previous publications [48, 47]. A total of 96 % cases were classified correctly using only this single parameter. The probability of an APA increases by a factor of 5.2 per percentage point of Ki67 immunoreactive nuclei (*p* < 0.001). A strong connection between Ki-67 LI, proliferation and relapse status of adenomas was confirmed in a recently published case-control study (*n* = 410) analyzing a post-surgical follow-up period of eight years [49]. In this and several follow-up studies by the same authors, a classification system for PA was proposed according to tumor size, type and a specific grade newly introduced [50, 51]. Although this data requires further verification by other groups and is currently not yet part of the WHO classification, it may represent a better analytical option for clinicians making decisions regarding the appropriate therapeutic management.

Invasive pituitary adenomas were described as being more aggressive in biological behavior and showing an increased growth rate compared to that of non-invasive tumors [36, 48, 52]. However, more than 47 % (*n* = 68/145) of the TPAs in our cohort showed an invasive growth pattern, a finding that is in line with several other observations published before (Table 3) [53, 6]. A low specificity (53 %) reflects the fact that invasive growth is not limited to the group of APA. According to our results, invasiveness was the least effective parameter for differentiating between both adenoma subtypes. It showed an especially broad confidence range (3.66; 18.42) [54]. Within both subgroups, only 64 % of the invasive adenomas were classified correctly. On the other hand, invasive growth remains a decisive prognostic factor in predicting patients’ disease-free status and overall outcome [49, 25]. This was not the subject of the present study due to incomplete follow-up data.

In order to describe additional criteria for the diagnosis of an APA, we analyzed the expression patterns of the alpha-subunit of glycoproteins (α-subunit) and the S-100 protein in the two subgroups. Both values (α-subunit *p* = 0.955; S-100 *p* = 0.269) were not helpful in the differential diagnosis and there were no significant differences between both subgroups (LRA). However, we found a significant correlation concerning the existence or absence of visible nucleoli and the correct diagnosis. Therefore, nucleoli are related significantly to the diagnosis of TPA (*p* = 0.008; OR = 0.4), a result which is contrary to other tumor entities like meningiomas [55, 5].

The diagnosis of APAs was introduced to describe a possible precursor lesion for pituitary carcinomas (PCAs), the only primary malignant tumor entity arising in the sellar region [56]. An early diagnosis of PCA is essential as the prognosis is usually poor (survival rate <1 year) [57, 58]. Due to the fact that PCA have no known histomorphological hallmarks, the diagnosis is still based on detectable metastases [59–64, 56]. To elucidate whether one or a combination of KI67 LI, p53 expression, invasive tumor growth and mitotic index are helpful in the diagnosis, 10 PCA cases were included in the study. All PCAs showed an invasive growth pattern. In combination with an elevated Ki-67 LI (Accuracy: 86 %; Specificity: 93 %), these are good prognostic markers for PCAs, as was previously suggested in other studies [58, 65–67, 48]. Absence of invasiveness in slowly growing TPAs largely reduces the likelihood of PCA development because it was already shown that PCAs do not develop a priori, but rather through malignant transformations of TPAs in the majority of cases [39, 65, 58].

A strong nuclear p53 expression was also a reliable (Accuracy: 86 %) and specific single marker (Specificity: 93 %) like Ki67 for PCAs and may explain their aggressive biological behavior [29, 68]. Interestingly enough, no mutations of the p53 gene were found in PCAs [56, 69, 70]. The OR for p53 (*p* = 0.01) of 1.8 is just as high as that of Ki-67 LI (*p* = 0.012), meaning that the growth rate is a very important characteristic in the analysis of clinical behavior, giving clinicians vital clues about aggressive tumors that are difficult to treat [48]. Only the mitotic index failed to demonstrate significant difference between the group of PCA and both other subtypes (LRA: *p* = 0.124; Point-biserial: *p* = 0.097). Despite these results, it must be noted that 6 PCA specimens (60 %) could not be reevaluated with respect to the number of mitoses. Therefore, the diagnostic relevance of the mitotic index should not be disregarded as irrelevant, especially due to its sensitivity rate of 100 %. Other studies confirmed this with similar findings of increased mitotic indices in progressive and metastasized tumors [71–73].

Comparing APAs and PCAs, there were no significant differences in LRA between the two groups with regard to Ki-67 Li (*p* = 0.223), p53 (*p* = 0.585), mitotic index (*p* = 0.274) and invasiveness (*p* > 0.999). Therefore, metastases remain the only reliable sign indicating PCAs (Tables 2 and 3) [59–64, 56].

An accurate classification of APAs is especially important so that an early diagnosis can be followed up with the best-possible therapy for the patients (careful watch, surgery, radiotherapy, medication, chemotherapy) [74–76]. With the cut-off values demonstrated here, a more precise categorization of adenomas in terms of their Ki-67, p53, mitotic rate and invasiveness values may be possible. In addition, the cut-off values also simplify the pathological analysis of adenomas in standard procedure, making the important comparison of various case study groups possible [77]. A prerequisite for this new opportunity, however, is that this method for diagnosing APAs is regularly applied across the board [24] so that clinical follow-up studies featuring large cohorts of APAs can be performed. Such studies would make it possible to further investigate whether the cell behavior correlates with the original diagnosis in terms of aggressiveness, proliferation, recurrence rate and the disease-free, post-surgery period. The clinical data sets of this study, which are to be further analyzed in a follow-up, provide the basis for additional studies of this kind.

## Conclusion

The newly defined cut-off values of the mitotic index (≥2) and p53 (≥2 %) makes the diagnosis of atypical adenomas (APAs) more reliable than was the case in the past. It is now possible to classify APAs in a standardized, more uniform manner. This, in turn, greatly increases the interrater reliability and also makes a direct comparison with similar studies much simpler. In addition, the accurate classification of APAs allows further studies including clinical follow up data to test the applicability or non- applicability of such a diagnosis according to treatment and/or prognostic values. According to this study, the best marker for differentiating typical pituitary adenomas and APAs is a Ki-67 (MIB-1) LI >4 % (Youden index: 0.92; AUC: 0.98).

## Additional files

Additional file 1: Table S1. Results of the correlation analysis of Ki-67 (%), p53 (%), number of mitosis in 10 HPF and invasive tumor growth in relation to the subgroups under study (TPA/APA/PCA). Abbreviations: N, number. (XLSX 10 kb) Additional file 2: Table S2. Results of the correlation analysis of Ki-67 (%), p53 (%), number of mitosis in 10 HPF and invasive tumor growth in relation to each other. Abbreviations: N, number. (XLSX 9 kb)

## References

[1] Buurman H, Saeger W (2006). Subclinical adenomas in postmortem pituitaries: classification and correlations to clinical data. Eur J Endocrinol.

[2] Daly AF, Rixhon M, Adam C, Dempegioti A, Tichomirowa MA, Beckers A (2006). High prevalence of pituitary adenomas: a cross-sectional study in the province of Liege, Belgium. J Clin Endocrinol Metab.

[3] Ezzat S, Asa SL, Couldwell WT, Barr CE, Dodge WE, Vance ML, McCutcheon IE (2004). The prevalence of pituitary adenomas: a systematic review. Cancer.

[4] Asa SL (2011) Tumors of the Pituitary Gland. American Registry of Pathology in collaboration with the Armed Forces Institute of Pathology in collaboration with the Armed Forces Institute of Pathology, AFIP, Washington DC. 1:275;ISBN 9781933477152.

[5] DeLellis RA (2004) Pathology and Genetics of Tumours of Endocrine Organs. Lyon, IARC Press. ISBN 9283224167.

[6] Saeger W, Ludecke DK, Buchfelder M, Fahlbusch R, Quabbe HJ, Petersenn S (2007) Pathohistological classification of pituitary tumors: 10 years of experience with the German Pituitary Tumor Registry. Eur J Endocrinol 156(2):203–216. doi:10.1530/eje.1.02326. 10.1530/eje.1.02326 17287410

[7] Al-Shraim M, Asa SL (2006). The 2004 World Health Organization classification of pituitary tumors: what is new?. Acta Neuropathol.

[8] Zada G, Woodmansee WW, Ramkissoon S, Amadio J, Nose V, Laws ER (2011). Atypical pituitary adenomas: incidence, clinical characteristics, and implications. J Neurosurg.

[9] Yildirim AE, Divanlioglu D, Nacar OA, Dursun E, Sahinoglu M, Unal T, Belen AD (2013). Incidence, hormonal distribution and postoperative follow up of atypical pituitary adenomas. Turk Neurosurg.

[10] Breslow NE, Day NE (1980). Statistical methods in cancer research. Volume I - The analysis of case-control studies. IARC Sci Publ.

[11] Menard S (2002) Applied Logistic Regression Analysis. vol Bd. 106;Bd. 2002. SAGE Publications. CA, USA; ISBN: 9780761922087.

[12] Youden WJ (1950). Index for rating diagnostic tests. Cancer.

[13] Hanley JA, McNeil BJ (1982). The meaning and use of the area under a receiver operating characteristic (ROC) curve. Radiology.

[14] Swets JA (1988). Measuring the accuracy of diagnostic systems. Science.

[15] Szumilas M (2010). Explaining odds ratios. Journal of the Canadian Academy of Child and Adolescent Psychiatry = Journal de l’Academie canadienne de psychiatrie de l’enfant et de l’adolescent.

[16] Nagelkerke NJ (1991). A note on a general definition of the coefficient of determination. Biometrika.

[17] Shtatland ES, Kleinman K, Cain EM (2002) One more time about R2 measures of fit in logistic regression. NESUG 15 Proceedings:222–226. Harvard Pilgrim Health Care & Harvard Medical School, Boston, USA.

[18] Cohen J, Cohen P, West SG, Aiken LS (2013) Applied Multiple Regression/Correlation Analysis for the Behavioral Sciences. Taylor & Francis. Lawrence Erlbaum Associates, Inc., Publishers. Mahwah, New Jersey; ISBN-13: 978-0805822236.

[19] Metz CE (1978). Basic principles of ROC analysis. Semin Nucl Med.

[20] Figarella-Branger D, Trouillas J (2006). The new WHO classification of human pituitary tumors: comments. Acta Neuropathol.

[21] Kontogeorgos G (2006). Predictive markers of pituitary adenoma behavior. Neuroendocrinology.

[22] Kontogeorgos G (2006). Innovations and controversies in the WHO classification of pituitary adenomas. Acta Neuropathol.

[23] Laws ER, Lopes MB (2006). The new WHO classification of pituitary tumors: highlights and areas of controversy. Acta Neuropathol.

[24] Perry A, Scheithauer BW (2006). Commentary: Classification and grading of pituitary tumors. Observations of two working neuropathologists. Acta Neuropathol.

[25] Wolfsberger S, Knosp E (2006). Comments on the WHO 2004 classification of pituitary tumors. Acta Neuropathol.

[26] Sumi T, Stefaneanu L, Kovacs K, Asa S, Rindi G (1993). Immunohistochemical study of p53 protein in human and animal pituitary tumors. Endocr Pathol.

[27] Gandour-Edwards R, Kapadia SB, Janecka IP, Martinez AJ, Barnes L (1995). Biologic markers of invasive pituitary adenomas involving the sphenoid sinus. Mod Pathol.

[28] Buckley N, Bates AS, Broome JC, Strange RC, Perrett CW, Burke CW, Clayton RN (1995). P53 protein accumulates in Cushings adenomas and invasive non-functional adenomas. J Clin Endocrinol Metab.

[29] Thapar K, Scheithauer BW, Kovacs K, Pernicone PJ, Laws ER (1996). p53 expression in pituitary adenomas and carcinomas: correlation with invasiveness and tumor growth fractions. Neurosurgery.

[30] Levy A, Hall L, Yeudall WA, Lightman SL (1994). p53 gene mutations in pituitary adenomas: rare events. Clin Endocrinol.

[31] Muller W, Saeger W, Wellhausen L, Derwahl KM, Hamacher C, Ludecke DK (1999). Markers of function and proliferation in non-invasive and invasive bi- and plurihormonal adenomas of patients with acromegaly: an immunohistochemical study. Pathol Res Pract.

[32] Lubke D, Saeger W, Ludecke DK (1995). Proliferation Markers and EGF in ACTH-Secreting Adenomas and Carcinomas of the Pituitary. Endocr Pathol.

[33] Oliveira MC, Marroni CP, Pizarro CB, Pereira-Lima JF, Barbosa-Coutinho LM, Ferreira NP (2002). Expression of p53 protein in pituitary adenomas. Brazilian journal of medical and biological research = Revista brasileira de pesquisas medicas e biologicas / Sociedade Brasileira de Biofisica [et al.].

[34] Saeger W (2005). Pituitary tumors: prognostic indicators. Endocrine.

[35] Madsen H, Borges TM, Knox AJ, Michaelis KA, Xu M, Lillehei KO, Wierman ME, Kleinschmidt-DeMasters BK (2011). Giant pituitary adenomas: pathologic-radiographic correlations and lack of role for p53 and MIB-1 labeling. Am J Surg Pathol.

[36] Hentschel SJ, McCutcheon IE, Moore W, Durity FA (2003). P53 and MIB-1 immunohistochemistry as predictors of the clinical behavior of nonfunctioning pituitary adenomas. Can J Neurol Sci.

[37] Bassarova AV, Popov AA (1998). Immunohistochemical detection of p53--effect of fixation and methods of antigen retrieval. Folia Histochem Cytobiol.

[38] Salehi F, Agur A, Scheithauer BW, Kovacs K, Lloyd RV, Cusimano M (2009). Ki-67 in pituitary neoplasms: a review--part I. Neurosurgery.

[39] Scheithauer BW, Gaffey TA, Lloyd RV, Sebo TJ, Kovacs KT, Horvath E, Yapicier O, Young WF, Meyer FB, Kuroki T, Riehle DL, Laws ER (2006). Pathobiology of pituitary adenomas and carcinomas. Neurosurgery.

[40] Dubois S, Guyetant S, Menei P, Rodien P, Illouz F, Vielle B, Rohmer V (2007). Relevance of Ki-67 and prognostic factors for recurrence/progression of gonadotropic adenomas after first surgery. Eur J Endocrinol.

[41] Hsu CY, Guo WY, Chien CP, Ho DM (2010). MIB-1 labeling index correlated with magnetic resonance imaging detected tumor volume doubling time in pituitary adenoma. Eur J Endocrinol.

[42] Gejman R, Swearingen B, Hedley-Whyte ET (2008). Role of Ki-67 proliferation index and p53 expression in predicting progression of pituitary adenomas. Hum Pathol.

[43] Honegger J, Prettin C, Feuerhake F, Petrick M, Schulte-Monting J, Reincke M (2003). Expression of Ki-67 antigen in nonfunctioning pituitary adenomas: correlation with growth velocity and invasiveness. J Neurosurg.

[44] Mastronardi L, Guiducci A, Spera C, Puzzilli F, Liberati F, Maira G (1999). Ki-67 labelling index and invasiveness among anterior pituitary adenomas: analysis of 103 cases using the MIB-1 monoclonal antibody. J Clin Pathol.

[45] Losa M, Franzin A, Mangili F, Terreni MR, Barzaghi R, Veglia F, Mortini P, Giovanelli M (2000). Proliferation index of nonfunctioning pituitary adenomas: correlations with clinical characteristics and long-term follow-up results. Neurosurgery.

[46] Paek KI, Kim SH, Song SH, Choi SW, Koh HS, Youm JY, Kim Y (2005). Clinical significance of Ki-67 labeling index in pituitary macroadenoma. J Korean Med Sci.

[47] Righi A, Agati P, Sisto A, Frank G, Faustini-Fustini M, Agati R, Mazzatenta D, Farnedi A, Menetti F, Marucci G, Foschini MP (2012). A classification tree approach for pituitary adenomas. Hum Pathol.

[48] Thapar K, Kovacs K, Scheithauer BW, Stefaneanu L, Horvath E, Pernicone PJ, Murray D, Laws ER (1996). Proliferative activity and invasiveness among pituitary adenomas and carcinomas: an analysis using the MIB-1 antibody. Neurosurgery.

[49] Trouillas J, Roy P, Sturm N, Dantony E, Cortet-Rudelli C, Viennet G, Bonneville JF, Assaker R, Auger C, Brue T, Cornelius A, Dufour H, Jouanneau E, Francois P, Galland F, Mougel F, Chapuis F, Villeneuve L, Maurage CA, Figarella-Branger D, Raverot G, members of H, Barlier A, Bernier M, Bonnet F, Borson-Chazot F, Brassier G, Caulet-Maugendre S, Chabre O, Chanson P, Cottier JF, Delemer B, Delgrange E, Di Tommaso L, Eimer S, Gaillard S, Jan M, Girard JJ, Lapras V, Loiseau H, Passagia JG, Patey M, Penfornis A, Poirier JY, Perrin G, Tabarin A (2013) A new prognostic clinicopathological classification of pituitary adenomas: a multicentric case-control study of 410 patients with 8 years post-operative follow-up. Acta Neuropathol 126(1):123–135. doi:10.1007/s00401-013-1084-y. 10.1007/s00401-013-1084-y 23400299

[50] Trouillas J (2014). In search of a prognostic classification of endocrine pituitary tumors. Endocr Pathol.

[51] Raverot G, Jouanneau E, Trouillas J (2014). Management of endocrine disease: clinicopathological classification and molecular markers of pituitary tumours for personalized therapeutic strategies. Eur J Endocrinol.

[52] Buchfelder M, Fahlbusch R, Adams EF, Kiesewetter F, Thierauf P (1996). Proliferation parameters for pituitary adenomas. Acta Neurochir Suppl.

[53] Meij BP, Lopes MB, Ellegala DB, Alden TD, Laws ER (2002). The long-term significance of microscopic dural invasion in 354 patients with pituitary adenomas treated with transsphenoidal surgery. J Neurosurg.

[54] Shakespeare TP, Gebski VJ, Veness MJ, Simes J (2001). Improving interpretation of clinical studies by use of confidence levels, clinical significance curves, and risk-benefit contours. Lancet.

[55] de la Monte SM, Flickinger J, Linggood RM (1986). Histopathologic features predicting recurrence of meningiomas following subtotal resection. Am J Surg Pathol.

[56] Heaney AP (2011). Clinical review: pituitary carcinoma: difficult diagnosis and treatment. J Clin Endocrinol Metab.

[57] Pernicone PJ, Scheithauer BW, Sebo TJ, Kovacs KT, Horvath E, Young WF, Lloyd RV, Davis DH, Guthrie BL, Schoene WC (1997). Pituitary carcinoma: a clinicopathologic study of 15 cases. Cancer.

[58] Ragel BT, Couldwell WT (2004). Pituitary carcinoma: a review of the literature. Neurosurg Focus.

[59] Scheithauer BW (1984). Surgical pathology of the pituitary: the adenomas. Part II Pathol Annu.

[60] Branch CL, Laws ER (1987). Metastatic tumors of the sella turcica masquerading as primary pituitary tumors. J Clin Endocrinol Metab.

[61] Morita A, Meyer FB, Laws ER (1998). Symptomatic pituitary metastases. J Neurosurg.

[62] Doniach I (1992). Pituitary carcinoma. Clin Endocrinol.

[63] Kaltsas GA, Grossman AB (1998). Malignant pituitary tumours. Pituitary.

[64] Asa SL, Mete O (2013) A History of Pituitary Pathology. Endocrine pathology. doi:10.1007/s12022-013-9284-5. 10.1007/s12022-013-9256-9 23872913

[65] Kaltsas GA, Nomikos P, Kontogeorgos G, Buchfelder M, Grossman AB (2005). Clinical review: diagnosis and management of pituitary carcinomas. J Clin Endocrinol Metab.

[66] Ironside JW (2003). Best practice No 172: pituitary gland pathology. J Clin Pathol.

[67] Suhardja A, Kovacs K, Rutka J (2001). Genetic basis of pituitary adenoma invasiveness: a review. J Neurooncol.

[68] Tanizaki Y, Jin L, Scheithauer BW, Kovacs K, Roncaroli F, Lloyd RV (2007). P53 gene mutations in pituitary carcinomas. Endocr Pathol.

[69] Pei L, Melmed S, Scheithauer B, Kovacs K, Benedict WF, Prager D (1995). Frequent loss of heterozygosity at the retinoblastoma susceptibility gene (RB) locus in aggressive pituitary tumors: evidence for a chromosome 13 tumor suppressor gene other than RB. Cancer Res.

[70] Pei L, Melmed S, Scheithauer B, Kovacs K, Prager D (1994). H-ras mutations in human pituitary carcinoma metastases. J Clin Endocrinol Metab.

[71] Thapar K, Yamada Y, Scheithauer B, Kovacs K, Yamada S, Stefaneanu L (1996). Assessment of mitotic activity in pituitary adenomas and carcinomas. Endocr Pathol.

[72] Sanno N, Teramoto A, Osamura RY, Horvath E, Kovacs K, Lloyd RV, Scheithauer BW (2003). Pathology of pituitary tumors. Neurosurg Clin N Am.

[73] Roncaroli F, Nose V, Scheithauer BW, Kovacs K, Horvath E, Young WF, Lloyd RV, Bishop MC, Hsi B, Fletcher JA (2003). Gonadotropic pituitary carcinoma: HER-2/neu expression and gene amplification. Report of two cases. J Neurosurg.

[74] Losa M, Mazza E, Terreni MR, McCormack A, Gill AJ, Motta M, Cangi MG, Talarico A, Mortini P, Reni M (2010). Salvage therapy with temozolomide in patients with aggressive or metastatic pituitary adenomas: experience in six cases. Eur J Endocrinol.

[75] Kaltsas GA, Mukherjee JJ, Plowman PN, Monson JP, Grossman AB, Besser GM (1998). The role of cytotoxic chemotherapy in the management of aggressive and malignant pituitary tumors. J Clin Endocrinol Metab.

[76] Colao A, Grasso LF, Pivonello R, Lombardi G (2011). Therapy of aggressive pituitary tumors. Expert Opin Pharmacother.

[77] Grossman AB (2006). The 2004 World Health Organization classification of pituitary tumors: is it clinically helpful?. Acta Neuropathol.

