# Erratum: Histological criteria for atypical pituitary adenomas – data from the German pituitary adenoma registry suggests modifications

**DOI:** 10.1186/s40478-016-0290-y

**Published:** 2016-02-29

**Authors:** Christian P. Miermeister, Stephan Petersenn, Michael Buchfelder, Rudolf Fahlbusch, Dieter K. Lüdecke, Annett Hölsken, Markus Bergmann, Ulrich Johannes Knappe, Volkmar H. Hans, Jörg Flitsch, Wolfgang Saeger, Rolf Buslei

**Affiliations:** 1 Departments of Neuropathology, Friedrich-Alexander University Erlangen-Nürnberg (FAU), Schwabachanlage 6, 91054 Erlangen, Germany; 2 ENDOC Center for Endocrine Tumors, Hamburg & University of Duisburg-Essen, Essen, Germany; 3 Departments of Neurosurgery, Friedrich-Alexander University Erlangen-Nürnberg (FAU), Erlangen, Germany; 4 Department of Neurosurgery, International Neuroscience Institute, Hannover, Germany; 5 Departments of Neurosurgery, University Clinic Hamburg-Eppendorf, Hamburg, Germany; 6 Department of Neuropathology, Klinikum Bremen Mitte, Bremen, Germany; 7 Department of Neurosurgery, Johannes Wesling Hospital Minden, Minden, Germany; 8 Department of Pathology, Ruhr University Bochum, Bochum, Germany; 9 Departments of Neuropathology, University Clinic Hamburg-Eppendorf, Hamburg, Germany

The original version of this article [1] unfortunately contained a mistake in the author list.

The name of one co-author is written wrong in the final version of the article; Dr Hans Ulrich Knappe should be Ulrich Johannes Knappe.

The updated author list is provided below:

Christian P. Miermeister^1^, Stephan Petersenn^2^, Michael Buchfelder^3^, Rudolf Fahlbusch^4^, Dieter K. Lüdecke^5^, Annett Hölsken^1^, Markus Bergmann^6^, Ulrich Johannes Knappe ^7^, Volkmar H. Hans^8^, Jörg Flitsch^5^, Wolfgang Saeger^9^ and Rolf Buslei^1^*
